# Mini-implants in the palatal slope – a retrospective analysis of implant survival and tissue reaction

**DOI:** 10.1186/1746-160X-8-32

**Published:** 2012-11-16

**Authors:** Thomas Ziebura, Stefanie Flieger, Dirk Wiechmann

**Affiliations:** 1 Department of Orthodontics, University of Münster, Albert-Schweitzer-Campus 1, Münster 48149, Germany; 2 Department of Orthodontics, Medizinische Hochschule Hannover, Carl-Neuberg-Str. 1, Hannover 30625, Germany

## Abstract

**Background:**

To identify insertion procedure and force application related complications in Jet Screw (JS) type mini-implants when inserted in the palatal slope.

**Methods:**

Setting and Sample Population: The Department of Orthodontics, the University Hospital Münster. Forty-one consecutively started patients treated using mini-implants in the palatal slope. In this retrospective study, 66 JS were evaluated. Patient records were used to obtain data on the mode of utilization and complications. Standardized photographs overlayed with a virtual grid served to test the hypothesis that deviations from the recommended insertion site or the type of mechanics applied might be related to complications regarding bleeding, gingival overgrowth or implant failure.

**Results:**

Two implants (3%) were lost, and two implants (3%), both loaded with a laterally directed force, exhibited loosening while still serving for anchorage. Complications that required treatment did not occur, the most severe problem observed being gingival proliferation which was attributable neither to patients’ age nor to applied mechanics or deviations from the ideal implant position.

**Conclusions:**

The JS mini-implant is reliable for sagittal and vertical movements or anchorage purposes. Laterally directed forces might be unfavorable. The selection of implant length as well as the insertion procedure should account for the possibility of gingival overgrowth.

## Background

Potential sites for mini-implant insertion in the maxilla comprise interradicular space, the infrazygomatic crest and the hard palate [1-4].

In terms of skeletal anchorage, the anterior hard palate is especially advantageous since root damage is very unlikely in this area. Furthermore, it provides good bony support [1,5-7]. Median and paramedian insertion as well as various mechanics have been described [4,8-13].

**Figure 1:**
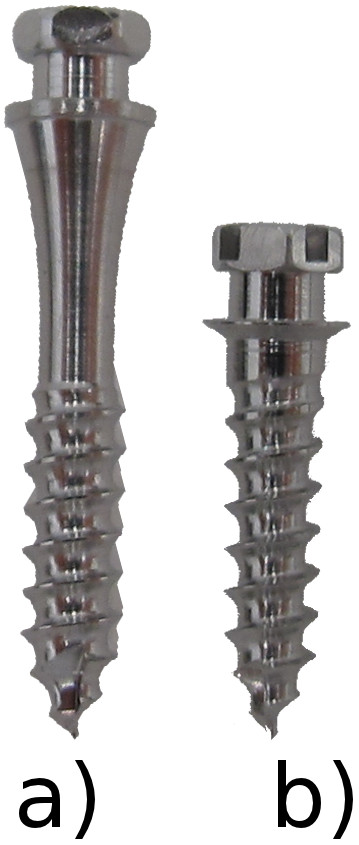
a) Jet Screw (JS, 5 mm neck); b) conventional mini-implant (Dual Top Anchor Screw, 8 mm long, 2 mm thread).

**Figure 2:**
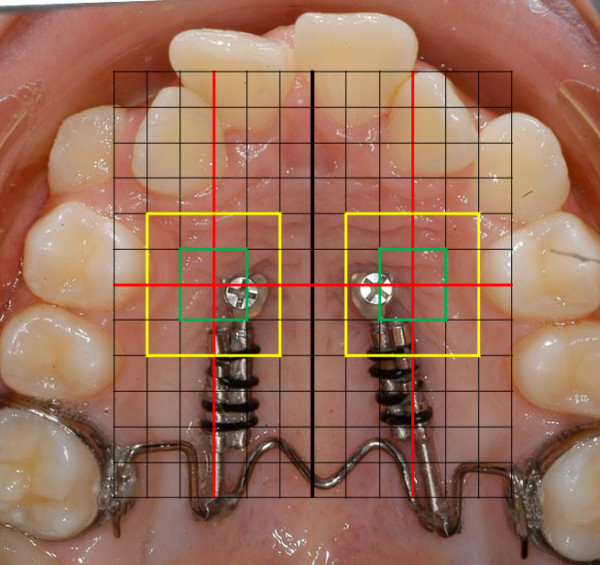
assessment of implant position (in this example the JS presents in association with the TopJet Distalizer) : green perimeter = desired position; yellow perimeter = slight deviation.

**Figure 3:**
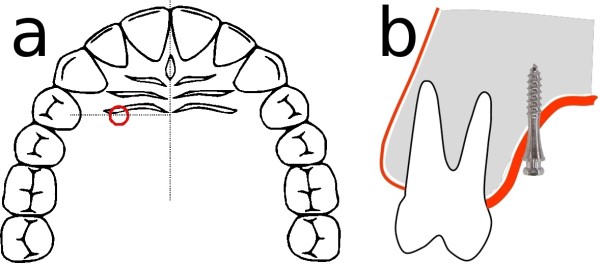
a) recommended insertion position; b) Jet Screw insertion angle.

The Jet Screw (JS) type mini-implant (Figure 1a, Promedia Medizintechnik GmbH, Siegen, Germany) was developed for insertion in areas with thick soft tissue such as the palatal slope. It is advertised for use with the TopJet Distalizer (H. Winsauer, Bregenz, Austria; Promedia Medizintechnik GmbH, Siegen, Germany, Figure 2). However, its applications in the department of orthodontics at the Münster university hospital comprise other types of mechanics, e. g. mesialization, indirect anchorage in extraction cases, vertical and transversal movements. It is recommended by the manufacturer to place the JS in the position which is determined as half of the distance of the perpendicular line segment from the raphe to the palatal cusp tip of the first bicuspid (Figure 3a).

The specified position offers good bony support [5,7,14]. Compared to other palatal insertion sites, it involves less distance between mini-implant and posterior teeth. As literature indicates, not only the recommended site itself offers sufficient bone for mini-implant insertion. Surrounding areas appear suitable as well [1,7]. This suggests that slight deviations might be tolerated. The insertion position may be varied to accommodate individual conditions, e. g. palatally impacted canines.

The oblique insertion of the JS (Figure 3b) – which is a result of uprighting the initially perpendicularly placed screw after a few revolutions resulting in an vertically oriented implant - can be expected to have no undesirable effects [5,7,14,15] but a medially faced part of the implant that is not gingivally covered while the laterally faced part has extensive gingival contact.

Most of the screw types available today feature a cylindrical or conic thread and a neck to accommodate the gingiva. In some mini-implants, a gingival collar is used to prevent overgrowth. However, such collars as featured in Figure 1b are adapted to parallel contact with soft tissue. Thus, they function best when the screw is inserted perpendicularly.

The JS features a long neck which widens towards the implant head. This design was concepted to be less prone to accumulating plaque and debris while also reducing gingival overgrowth.

The aim of this work was to retrospectively assess if there are any complications to be expected from insertion in the recommended location or from respective deviations and if the mode of loading influences implant survival.

## Methods

All patients in which JS were consecutively inserted in the time between December 2009 and November 2011 by either TZ or SF were included in this study. Exclusion criteria were disagreement to scientific usage of clinical photographs by either the parents or patients themselves. Also, patients who aborted treatment were excluded from the study. The present retrospective study was not based on experimental research carried out on humans or animals. Therefore, an approval of an ethics committee was not necessary. Written informed consent was obtained from the patient for publication of this report and any accompanying images.

### Implants and insertion procedure

The screws investigated in this study had necks of 3 or 5 mm length for accommodation of different gingival thicknesses. The thread is 8 mm long and 2 mm in diameter.

All mini-implants were inserted by the same protocol:

• Informed consent regarding potential risks, complications and behavior via standardized documents

• surface anesthesia with 1% lidocaine spray applied using a cotton ball

• infiltration using 4% articaine solution

• mouth rinse with 2% chlorhexidine digluconate

• assessment of gingival thickness using a probe

• choice of neck length: 3 mm neck for gingival thicknesses not exceeding 3.5 mm

• insertion using a surgical handpiece

### Insertion area

For determining the implant position, intraoral photographs were used that had been fabricated by one experienced, professional photographer using a Nikon D100 camera and a mirror in order to obtain an orthogonal view of the dental arch and the palate (SB-29s flashlight, aperture f/32, 1/100 s exposure time). All pictures were independently screened by TS for JSs in another than the recommended position, a procedure that was repeated two weeks apart to ensure data correctness. To permit for a semi-quantitative determination, the photographs were overlaid with a grid whose dimensions were established by separating the distance between the palatal cusps of the first bicuspids into twelve equal parts (Figure 2). Superimposition of an equally dimensioned set of lines, rotated by 90°, yields a regularly spaced grid with cell dimensions corresponding approximately to the size of an implant head. No deviation from the recommended position was assumed when a screw was displayed within the green perimeter in Figure 2. A deviation was assumed to be slight when it covered less than the dimensions of one grid cell horizontally and vertically (yellow perimeter). All other deviations were deemed severe (beyond yellow perimeter). In cases where an implant head appeared between two cells, it was attributed to the cell where the larger part of the implant head was displayed.

### Measurement error

Six JSs (9%) were temporarily left in situ after removal of the respective mechanics in case of later anchorage requirements. These unloaded implants were repeatedly photographed on subsequent appointments, and the photographs served to test the reliability of the measurement procedure. Mean coordinates were calculated from all measured positions for each of the six JSs. The sagittal and transversal distances between each individual measurement and the mean coordinates were established.

### Complications

Patient files served for identification of JS loosening or loss and bleeding complications at the time of insertion and during a follow-up period of 6 months.

### Mechanics

Patient records and pictures served to identify and evaluate various devices and constructions based on JSs as well as the force vectors delivered by these appliances.

### Statistics

Data was entered into an SPSS file (SPSS Version 19, IBM Corporation, Armonk, USA) by TZ and checked for correctness in all cases by SF.

## Results

### Patients

N = 0 patients/parents disagreed to scientific usage of clinical photographs, n = 1 aborted treatment and was thus excluded.

66 JSs, consecutively inserted by both authors in 41 patients (19 male, 22 female, mean age 15.1 years) met the inclusion criteria.

Patient age ranged from 10 to 37 years (mean 15.1 years, SD 4.9).

Notably, three patients (7.3%) had a cleft palate on the side where a screw was inserted.

Sixteen (39%) of the 41 patients received 1 JS, in 25 cases (61%) two JSs were inserted. In one of these cases (2.4%), both JSs were inserted ipsilaterally.

### Insertion area

Two implant positions (3%) could not be assessed reliably due to gingival overgrowth (Additional file 1: Table S1). In 36 implants (54.5%), no deviation from the recommended insertion site was found (i. e. the implant head’s position was within green perimeter in Figure 2). In another 18 (27.3%) the deviations were discrete (within the yellow perimeter). Ten screws (15.2%) exhibited larger deviations (beyond the yellow perimeter). Some of the deviations from the insertion area were due to sagittal adjustments made to obtain favorable activation distances for the respective mechanics (n = 13, 19.7%). Others were conditioned by palatally impacted teeth (n = 3, 4.5%). Furthermore, some unintentional deviations were caused by the slope of the palate leading to slipping of the implants insertion (n = 9, 13.6%).

### Measurement error

The measurements on six unloaded JSs yielded a mean sagittal deviation amounting to 20% of a cell width and a mean transversal deviation of 10% of a cell width (Table 1).

**Table 1:** Repeated measurements (T1-T4) in unloaded screws (u1-u6); transversal and sagittal deviations (in fractions of a cell width) from median coordinates

		**u1**	**u2**	**u3**	**u4**	**u5**	**u6**
**T1**	transversal deviation	0,18	0,115	0,14	0,06	0	0
	sagittal deviation	0,2	0,03	0	0,29	0,03	0,02
**T2**	transversal deviation	0	0,035	0	0	0,01	0,1
	sagittal deviation	0,2	0,34	0,8	0,54	0	0
**T3**	transversal deviation	0,22	0,035	0,17	0,17	0,19	0,34
	sagittal deviation	0	0,03	0	0	0,45	0,39
**T4**	transversal deviation		0,185				
	sagittal deviation		0,15				

### Complications

Two implants in one patient were lost (3%). These were located in the recommended position. The patient admitted – despite opposite instructions after implant insertion – having developed a habit of manipulating the implants with her fingers, which is estimated to be the reason for failure.

One implant (1.5%), latero-ventrally located and loaded with a laterally-directed force exhibited loosening. Also, in one cleft-palate-patient (1.5%), with a Jet Screw located at the recommended site loosening did occur. Notably, this implant was loaded with a medially-directed force. Both of these implants remained in use and served their purpose.

Mild bleeding (defined as a small accumulation of blood limited to the area adjacent to the implant) during insertion was common (n = 28 cases, 68.3%) but stopped during the insertion time of the required mechanics thus not requiring any specific measures.

Overgrowth, defined as the partial or complete covering of the implant head by soft tissue, was the most common complication. In eight implants (12.1%) the screw head was covered by palatal mucosa. However, incisions or other surgical measures were not necessary since the implants were still accessible for removal using a spatula in the screws’ cross-recesses instead of a screw driver.

Five of the implants (7.6%) on which this complication occurred had been placed at the recommended site. One (1.5%) was located medially. In two (3%), the position could not be assessed accurately because the implant head was completely covered by mucosa. In six implants (9.1%) where overgrowth occurred, Jet Screws with 3 mm necks had been used.

### Mechanics

Table 2 displays the frequency at which different types of mechanics were applied. From a total of 41 patients, 17 (41.5%) were treated with a TopJet Distalizer (Figure 2).

**Table 2:** Patients’ age distribution, respective indications for JS insertion and number of patients exhibiting complications: a = gingival overgrowth, b = implant loosening, c = implant loss

**age**	**no. of patients**	**space closure**	**distalization**	**vertical movement**	**transverse movement**	**anchorage**
10	4	0	4	0	0	0
11	4	0	4 / a = 2	0	0	1
12	6	5	2	1	0	0
13	4	3 / a = 1	0	0	1 / b = 1	0
14	3	2	1	0	0	0
15	4	3	0	0	0	1
16	4	3 / c = 2	1	0	0	0
17	5	0	2	1	1 / b = 1	1
18	1	0	0	1 / a = 1	0	1
19	3	0	2 / a = 1	1	0	0
20	1	1	0	0	0	0
24	0	0	0	0	0	0
27	1	0	1	0	0	0
37	1	1	0	0	0	0
**total**	**41**	**18**	**17**	**4**	**2**	**4**

**Figure 4:**
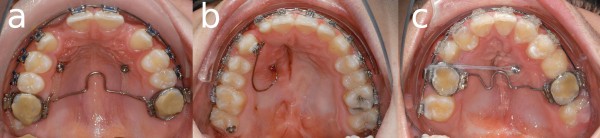
a) space closure mechanics; b) canine extrusion; c) transversal movement.

In 18 cases (43.9%), JSs served as anchorage for maxillary space closure and mesialization of the buccal segments. The mechanics applied in these cases consisted of a transpalatal arch and elastic chains connecting it with the JSs (Figure 4a). This approach provides a connection approximating the vertical level of the dental center of resistance.

Posterior anchorage was the treatment objective in four cases (9.8%). .016x.022 stainless steel wire segments connecting implants and first molars served this purpose.

**Figure 5:**
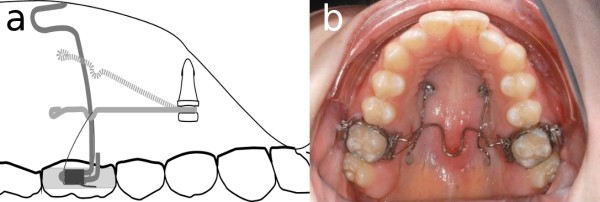
a) intrusion cantilever (view from palatal side); b) intrusion cantilevers inserted.

**Figure 6:**
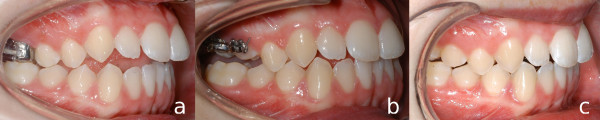
a) before intrusion; b) over-correction; c) start of treatment with lingual appliance.

Occasionally (n = 4, 9.8%), JSs were used to exert vertical control. To extrude canines (n = 2, 4.9%), a C-spring is fabricated and connected to implant and teeth (Figure 4b). Posterior intrusion can be achieved through distally extended cantilevers (Figures 5 and 6). Those serve as vertical anchorage for the first molars. A transpalatal arch is used to avoid palatal tipping.

In two cases (4.9%), the JSs were loaded with a transverse force to induce lateral or medial tooth movement (Figure 4c). Notably, both of these implants exhibited loosening.

## Discussion

### Patients

The mean age of patients in our study was younger and the gender distribution more balanced than in other studies [16,17]. Notably, three implants (4.5%) were inserted in cleft palate patients.

### Insertion area

The results of the present study indicate that the palatal slope offers good stability for mini-implants. Deviations from the predetermined position did not lead to complications in this investigation, but there were not enough deviating cases in order to ensure statistical validity to generalize this observation.In comparison to other studies with palatal implants, loosening and loss rate is comparably low [17,18].

### Measurement error

In contrast to e. g. three-dimensional measurement of the implant position on dental casts the method of assessment via standardized photographs has the disadvantage of being prone to slight measurement errors due to discrete tipping if the mirror. This possible risk has been minimized by performance of this process through a single and experienced photographer and standardized camera settings. Evaluation of dental casts would have been accompanied by the problems of tear off of alginate in the area of the JS thus leading to imprecise 3D measurement.

The repeated measurements (Table 1) imply that measurement errors average to 0.2 cell widths sagittally and 0.1 cell widths transversally. The possibility of changes in the implants’ position between individual measurements could be eliminated by using unloaded implants.

### Complications

Notable bleeding complications did not occur which may be due to the greater palatine artery’s diameter decreasing anteriorly [19]. This implies that pronounced distal deviations from the recommended insertion site should be avoided, although even those did not cause problems in the investigated cases.

Palatal mucosa thickness can easily exceed 3 mm which needs to be taken into account [20]. Overgrowth did not cause severe complications but could develop into a time-consuming annoyance. It might be avoided either by reducing insertion depth or by choosing implants with longer necks, the latter being preferable regarding primary and long-term stability.

### Mechanics

A variety of different mechanics was attached to JSs, benefiting from their lateral position and the associated proximity of the teeth to be moved. Laterally directed forces might be unfavorable, but more cases have to be treated and investigated in order to validate this suspicion.

## Conclusions

JS type mini-implants feature good clinical stability and do not cause severe complications. They offer safe and versatile anchorage for a large variety of mechanics. Laterally directed forces might be unfavorable but this hypothesis needs to be evaluated in more cases.

Deviations from the recommended insertion are unlikely to cause problems which might be favourable for unexperienced practitioners and in cases where deviations are necessary due to impacted teeth or clefts.

To avoid gingival overgrowth, screws with longer necks should be chosen in case of doubt. Also, patients need to be instructed to sustain good oral hygiene, regularly remove accumulations of plaque on the implant head and the secondary construction and avoid manipulations at the implant.

## Competing interests

The authors declare that they have no competing interests.

## Authors’ contributions

TZ initiated the investigation leading to these results; participated in discussions on the undertaking of the study; conceived, designed, and supervised the study; interpreted the data; reviewed all iterations of the paper; and wrote the first draft and the final version of the paper. SF participated in discussions on the undertaking of the study, designed and supervised the study, interpreted the data, reviewed the paper for content, and reviewed and contributed to the writing of all iterations of the paper, including the final version of the manuscript. DW participated in discussions on the undertaking of the study, supervised and did the statistical analysis, interpreted the data, reviewed the paper for content, and reviewed and contributed to the writing of all iterations of the paper, including the final version of the manuscript. All authors read and approved the final manuscript.

## Supplementary Material

Additional file 1 **Table S1.** Number of screws deviating from recommended insertion site, based on the grid in Figure 2: No deviation is defined as screw head being displayed within the green perimeter, severe deviations are defined as the screw head protruding beyond the yellow perimeter. (DOCX 12 kb) Click here for file
