# Supplementary material for: Mini-implants in the palatal slope – a retrospective analysis of implant survival and tissue reaction
Source: Head Face Med. 2012 Nov 16;8:32. doi: 10.1186/1746-160X-8-32 (PMC3546431; doi:10.1186/1746-160X-8-32)
Supplement: Additional file 1 — Table S1. Number of screws deviating from recommended insertion site, based on the grid in Figure 2: No deviation is defined as screw head being displayed within the green perimeter, severe deviations are defined as the screw head protruding beyond the yellow perimeter. (DOCX 12 kb) [file 1746-160X-8-32-S1.doc]

|  |  | **transverse deviations** | | | | |
| --- | --- | --- | --- | --- | --- | --- |
|  |  | **far-medial** | **medial** | **no deviation** | **lateral** | **far-lateral** |
| **sagittal deviations** | **far-ventral** | 0 | 0 | 0 | 0 | 0 |
|  | **ventral** | 2 | 1 | 0 | 1 | 0 |
|  | **no deviation** | 1 | 9 | 36 | 1 | 0 |
|  | **dorsal** | 0 | 3 | 3 | 0 | 0 |
|  | **far-dorsal** | 0 | 0 | 7 | 0 | 0 |

Table 1: Number of screws deviating from recommended insertion site, based on the grid in fig. 2: No deviation is defined as screw head being displayed within the green perimeter, severe deviations are defined as the screw head protruding beyond the yellow perimeter
